# Lingual Osseous Choristoma: A Systematic Review of Lesion Presentation, Histology, and Morphology

**DOI:** 10.51894/001c.17543

**Published:** 2020-10-30

**Authors:** Zaid J Shareef, Sarah J Shareef, Connor C Kerndt, Arielle Aughenbaugh, Anthony Di Ponio

**Affiliations:** 1 MSUCOM SCS/MSUCOM; 2 College of Human Medicine MSU; 3 Internal Medicine Spectrum Butterworth Hospital; 4 Otolaryngology Henry Ford Macomb Hospital

**Keywords:** surgical tongue resection, gagging/globus sensation, pedunculated tongue mass, osseous choristoma

## Abstract

**INTRODUCTION:**

Osseous choristomas of the tongue are rare, benign tumor-like lesions composed of abnormally placed bone and cartilage tissue. The few publications to date concerning this condition have been primarily limited to case reports. This systematic review aimed to clarify the clinical presentations of osseous choristomas and how to delineate them from other oral pathologies.

**METHODS:**

The authors utilized PubMed, Embase, and Cochrane Library reference databases from 1971 to mid-2020. Search terms were “osseous choristoma,” “oral cavity,” and “lingual.” Preferred Reporting Systems for Systematic Reviews and Meta-Analysis (PRISMA) guidelines were used to aggregate relevant data from each study. The authors specifically collected data regarding patient demographics, clinical findings, symptoms, treatments, and subsequent outcomes relating to lingual osseous choristomas.

**RESULTS:**

A total of 35 (14.6% of total identified) publications that met inclusion criteria were identified concerning a total of 69 lingual osseous choristoma cases. Results were compiled focusing on sex and age, presenting symptoms, histology, appearance of the lesion base being most commonly pedunculated (e.g., stalk or stem-like), the lesion’s location on the tongue, and subsequent treatments. Osseous choristomas had a higher rate of occurrence in females, 48 (70%) and those under the age of 40. Symptomatic presentations occurred in 38 (55%) patients, with the most common presenting symptoms being gagging/globus (i.e., lump or foreign body) sensation (n = 47, 68%) and dysphagia (n = 20, 29%). Identified masses were pedunculated in 33 (80%) of cases and eight (20%) were identified as sessile (i.e., immobile). A total of 41 (59%) lesions were more commonly located in the posterior one third of the tongue compared to 28 (41%) in the anterior two thirds of the tongue. Of those 49 (71%) cases requiring surgical mass excisions, recurrence was reported in 0% of cases.

**CONCLUSIONS:**

Although osseous choristomas are benign processes that rarely arise from the tongue, providers should carefully inspect patients with a gagging/globus sensation and pedunculated mass toward the back of the tongue. Surgical resection remains the best treatment to prevent recurrence.

## INTRODUCTION

A choristoma has been defined as a tumor-like growth of normal tissue in an ectopic (i.e., abnormal) location.^1^ Choristomas are seldom observed in the oral cavity and when identified, are more often composed of bone or cartilage.^2^ However, masses may also consist of peripheral glial tissue (i.e., connective tissue that supports neurons), sebaceous glands (i.e., exocrine glands that secrete oily sebum to aid in lubrication), or gastrointestinal tissue.^1,2^

Lingual (i.e., relating to, near, or on the side of the tongue) osseous (ossified; i.e., consisting of, or turned into, bone) choristomas are particularly rare entities, with fewer than 100 cases documented in the English literature since first defined in 1913.^3^ The pathogenesis of these benign lesions has remained quite ill-defined.^2^ These lesions are most commonly identified in female patients in the third and fourth decades of life.^3–5^ Although these lesions are considered self-limiting in growth, they can mimic malignant diseases processes that physicians should differentially diagnose when working up patients with lingual masses.^2–4^

On physical examination, lingual osseous choristomas frequently appear as painless, narrow based or pedunculated (i.e., stalk or stem) nodules on the tongue that are firm with palpation.^1,6^ These lesions are more commonly localized on the posterior aspect of the tongue, although a small number of reported osseous choristoma cases have occurred on lateral aspects of the tongue.^2,3,7^

Since the tongue is a distinct anatomically delicate structure containing multiple different innervations and landmarks, diagnosing abnormal tongue masses can be complex. Tongue masses can include osseous choristomas or other lesions such as squamous cell carcinomas (SCCs) of the tongue, hemangiomas (i.e., benign blood vessel tumors), or pyogenic granulomas (i.e., small round epithelial growths that also contain blood vessels and capillaries).^8,9^

Even though some patients may present asymptomatically, a wide array of symptoms including dysphagia (i.e., difficulty swallowing), foreign body sensation, gagging, discomfort, and pain have been reported. Although a thorough physical examination and imaging may assist in identifying the mass, a definitive diagnosis may require a biopsy and histologic examination tests.^2^

To the authors’ knowledge, only one prior systematic review (1971) has been previously published regarding this type of tongue lesion.^5^ The purpose of this current systematic review was to update providers’ clinical understanding of lingual osseous choristomas using data from the publications to date concerning patient demographic characteristics, treatment types and subsequent health outcomes associated with these types of lesions.

## METHODS

### Screening Process

**Figure 1:**
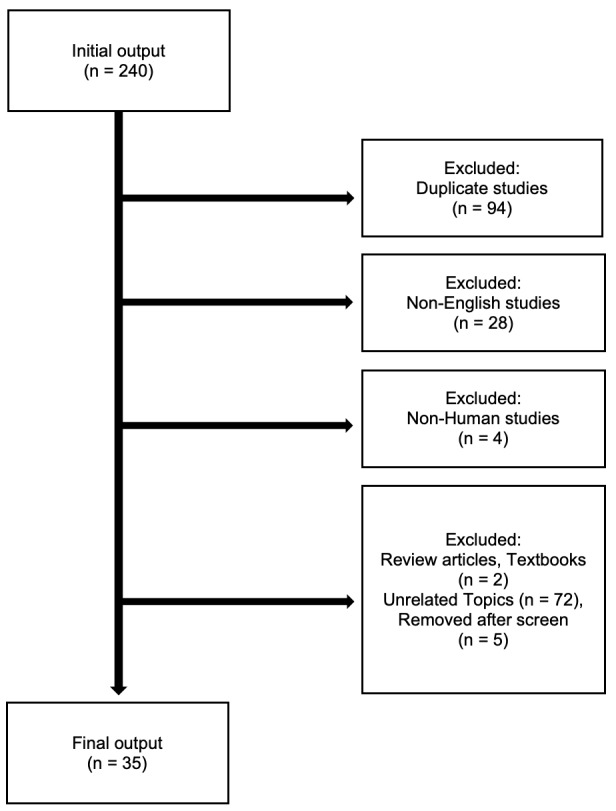
Overview of Inclusion criteria used for collection in this study

This systematic review was conducted observing the 2009 Preferred Reporting Systems for Systematic Reviews and Meta-Analysis (PRISMA) guidelines.^10^ The authors’ preliminary search extracted citations from Embase, PubMed, and Cochrane databases from 1971 until February, 2020. Search terms used during this review included “osseous choristoma,” “oral cavity,” and “lingual.” A total of 240 articles were initially identified for consideration. (Figure 1) Each article was reviewed by two independent authors (authors SJS and AA).

All duplicate and non-English studies were first removed from consideration as were non-human studies and non-original publications (e.g., textbooks and review articles). Publications were then evaluated if they met each of the following inclusion criteria: a) an Oxford Centre for Evidence-Based Medicine^11^ (OCEBM) classification level of IV or higher, b) articles specific to lingual origin lesions, and c) articles describing a histopathologic confirmation of osseous choristoma. Articles with sub-IV classification levels that were not patient case series or case reports were not considered. Details regarding further search and inclusion results are depicted in Figure 1.

### Data Extraction

After completing the initial article screening, data regarding patient demographics, presenting symptoms, mass location, imaging and histological findings, treatment and subsequent health outcomes were extracted and recorded by two independent reviewers (authors ZJS and CCK). Common clinical data patterns were sorted and placed into subgroups for visual data comparisons using Microsoft Excel (author ZJS).

## RESULTS

The authors’ initial literature review identified a total of 240 potential articles or abstracts from Embase, PubMed, and Cochrane databases. Of the 240 articles, 35 (14.6%) case series or case report studies met the inclusion criteria for this systematic review. In total, 69 discrete patients with lingual osseous choristoma were described in these 35 articles. One case report did not provide patient information but was included as able in summary reporting. Major details of each case included are compiled in Table 1, focusing on age, sex, symptom presentation, histology, lesion base, location, and clinical treatment.

### Demographics and Medical History Characteristics

**Figure 2:**
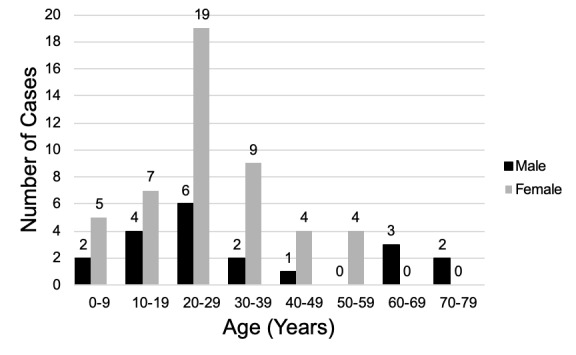
Histogram depicting crosstabulation of age and sex by decade of life.

The majority (48 or 70%) of patients with osseous choristomas were females. Patients’ age at time of presentation ranged from five through 73 years, with an average age of 28.1 (SD 16.7) years. Figure 2 provides a visual representation of these results, showing a crosstabulation of sex by age decade subgroup. Due to the smaller number of eligible articles we could find, no statistical comparisons were conducted to gauge the significance of subgroup variations.

Of all reported cases, only eight (11.6%) articles described patients with one or more of the following past medical conditions: Type I Diabetes, a mucocele (i.e., cyst-like space in the mouth that contains mucus), enlarged adenoids, hypertension, epilepsy, functional abdominal pain, iron deficiency anemia, or autism spectrum disorder.

### Symptomatology and Mass Location

**Figure 3:**
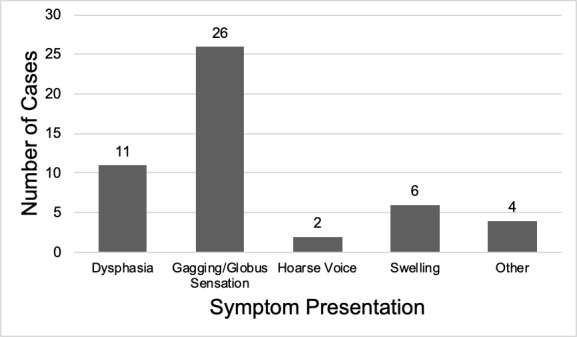
Symptomatology on presentation

Approximately 38 (55.07%) of patients had symptomatic presentations. Of these symptomatic patients, 26 (68%) reported experiencing a gagging/globus sensation. The next most common symptom included dysphagia (n =11, 28.9%), followed by tongue swelling (n = 6, 15%), pain (n = 3, 8.0%), and dysphonia (i.e., hoarseness/abnormal speech) (n = 2, 5.0%). Other symptomatic complaints described by one (1.5%) patient each included tongue discharge, breathing difficulties, nausea, restriction in tongue movement and irritation. Figure 3 displays symptom frequencies at time of presentation.

### Imaging and Histology

Of 69 reporting cases, imaging was performed in only 10 (14%) patients. Five (7%) cases reported radiographic imaging, three (4%) received computerized tomography (CT) imaging, and two (2%) patients underwent magnetic resonance imaging (MRI). On examination, the lesion morphologies were included in 41 (59%) of cases, of which a 33 (80%) were pedunculated and eight (20%) were sessile.

Histologic findings were reported in 49 (71%) of cases after lesion biopsy. Classification parameters included the presence of cartilage, stratified squamous epithelium covering, presence of osteocyte (i.e., bone cell) or osteoblast (i.e., cells that secrete the matrix for bone formation), and the presence of haversian canals (i.e., microscopic tubes in bones that allow blood vessels and nerves to travel through).

All 49 cases confirmed findings were consistent with mature compact bone. Of the total histological findings, stratified squamous epithelium covering was reported in 37 (75%) patients, followed by presence of osteocytes in 19 (38%) patients, haversian canals, 18 (36%), osteoblasts 24 (12%), and cartilage, two (4.8%).

### Location on Tongue and Management

Most lesions were reported on the top dorsal aspect of the tongue. Case locations were then stratified as anterior two thirds or posterior one third of the tongue. In 28 (41%) patients, lesions were found on the front anterior two thirds of the tongue, while 40 (59%) patients’ lesions were found on the posterior third of the tongue. A common specific location of lesions in 15 (21%) of cases was the posterior dorsum of the tongue at the apex of the sulcus terminalis (i.e., foramen cecum).^12^

The reported polarity of 14 (59%) of lesions was also examined. Lesions were most frequently located on the tongue midline, (n = 16, 39%) and exhibited equal distribution on the left and right side respectively, (n = 13, 31%). The most common intervention strategy completed in 49 (71%) of patients was surgical mass resection. Specific resection methods were outlined in 41 (84%) of cases, of which 38 patients (77%) underwent conventional surgical excision. Alternatively, two (4%) patients had surgical excision via a carbon dioxide laser, and one (2%) patient received electrosurgery.

**Table 1:** Descriptive Overview of Results

**Year**	**Author**	**LOE & Design**	**Decade of Age and Sex**	**Presenting Symptom**	**Histology**	**Base of Lesion**	**Location on Tongue**	**Treatment**
2020	Kutlesi, et. al.	4, CR	20s, F	G/FB/Mass	Ob, MCB	-	Anterior 2/3 and Posterior 1/3	Excision
2018	Macedo, et al.	4, CR	Child, M	Symptomatic	SSE, MCB	Sessile	Posterior 1/3	Excision
2017	Yoshimura, et al.	4, CR	Child, M	G/FB/Mass	SSE, Oc, Ob, MCB	Pedunculated	Posterior 1/3	Excision
2017	Heinz, et al.	4, CR	20s, F	G/FB/Mass	SSE	Pedunculated	Anterior 2/3	Laser/CO_2_
2016	Tran, et al.	4, CR	30s, F	D, G/FB/Mass	SSE, Oc, HC, MCB	Pedunculated	Posterior 1/3	Excision
2016	Davidson, et al.	4, CR	Child, M	D, G/FB/Mass	SSE, MCB	-	Posterior 1/3	Excision
2016	Adhikari, et al.	4, CS	Child, F	G/FB/Mass, S	SSE, MCB	Pedunculated	Left Posterior 1/3, Foramen Cecum	Excision
2016	Adhikar, et al.	4, CS	20s, F	Pain	SSE, MCB	Pedunculated	Right Posterior 1/3, Foramen Cecum	Excision
2016	Turan, et al.	4, CR	40s, F	G/FB/Mass, S	MCB	Sessile	Right Posterior 1/3, Foramen Cecum	Excision
2016	Candido, et al.	4, CR	Child, F	G/FB/Mass	MCB	Sessile	Left Anterior 2/3	Excision
2015	Standford, et al.	4, CR	Child, M	As	SSE, MCB	-	Left Posterior 1/3	Excision
2015	Girgis, et al.	4, CR	30s, F	G/FB/Mass	MCB	Pedunculated	Posterior 1/3, Foramen Cecum	Excision
2014	Yamamoto, et al.	4, CR	Child, M	D, H, S	SSE, Oc, MCB	Pedunculated	Right Anterior 2/3	Excision
2014	Gorini, et al.	4, CR	Child, F	G/FB/Mass	SSE, HC, MCB	Sessile	Midline Posterior 1/3, Foramen Cecum	Excision
2011	Liu, et al.	4, CR	Child, M	As	MCB	Pedunculated	Right Posterior 1/3	Excision
2008	Andressakis, et al.	4, CR	70s, M	D, S, Pain	Oc, MCB	-	Midline Posterior 1/3	Excision
2007	Demirseren, et al.	4, CR	20s, M	G/FB/Mass	C, MCB	-	Anterior 2/3	Excision
2007	Benamer, et al.	4, CR	Child, F	G/FB/Mass	SSE, Ob, MCB	Pedunculated	Midline Posterior 1/3	Excision
2000	Supiyaphun, et al.	4, CS	40s, F	As	SSE, Oc, MCB	Pedunculated	Left Anterior 2/3	Excision
2000	Supiyaphun, et al.	4, CS	Child, F	As	SSE, MCB	Pedunculated	Left Anterior 2/3	Excision
2000	Supiyaphun, et al.	4, CS	40s, F	D	SSE, MCB	Pedunculated	Left Anterior 2/3	Excision
1998	Lin, et al.	4, CR	20s, F	As	SSE, Oc, MCB	Pedunculated	Midline Anterior 2/3	Electric
1998	Vered, et al.	4, CS	40s, M	D, G/FB/Mass	Oc, MCB	-	Left Anterior 2/3	Excision
1998	Vered, et al.	4, CS	20s, M	G/FB/Mass, Pain	Oc, Ob, MCB	-	Midline Posterior 1/3	Excision
1996	Ngeow, et al.	4, CR	20s, F	As	SSE, MCB	Pedunculated	Midline Posterior 1/3, Foramen Cecum	Excision
1996	Manganaro	4, CR	N/A	As	-	-	-	-
1993	Ishikawa, et al.	4, CS	50s, F	G/FB/Mass	SSE, HC, MCB	Pedunculated	Midline Posterior 1/3, Foramen Cecum	Excision
1993	Ishikawa, et al.	4, CS	Child, F	As	SSE, MCB	Pedunculated	Midline Anterior 2/3	Excision
1989	Nash, et al.	4, CR	30s, M	As	MCB	-	Right Posterior 1/3	-
1988	West, et al.	4, CR	Child, F	D, G/FB/Mass	C, SSE, MCB	-	Foramen Cecum	Laser/CO2
1987	Tohill, et al.	4, CS	30s, F	As	SSE, Oc, HC, MCB	-	Left Anterior 2/3	Excision
1987	Tohill, et al.	4, CS	20s, F	As	C, SSE, MCB	-	Right Anterior 2/3	Excision
1987	Tohill, et al.	4, CS	60s, M	As	SSE, MCB	-	Left Posterior 1/3	Excision
1986	Cabbabe, et al.	4, CR	Child, F	As	SSE, Oc, HC, MCB	Pedunculated	Midline Posterior 1/3	Excision
1985	Weitzner	4, CS	50s, F	As	HC, MCB	-	Anterior 2/3	Excision
1985	Weitzner	4, CS	20s, F	As	HC, MCB	-	Posterior 1/3	Excision
1985	Weitzner	4, CS	20s, F	H	HC, MCB	-	Posterior 1/3	Excision
1984	Shimono, et al.	4, CS	40s, F	S	C, SSE, MCB	-	Midline Posterior 1/3	Excision
1984	Shimono, et al.	4, CS	30s, F	S	SSE, MCB	Pedunculated	Posterior 1/3, Foramen Cecum	Excision
1984	Sheridan	4, CS	20s, F	As	SSE, MCB	Pedunculated	Midline Anterior 2/3	Excision
1984	Sheridan	4, CS	60s, M	As	SSE, MCB	Pedunculated	Anterior 2/3	Excision
1984	Main	4, CR	50s, F	G/FB/Mass	-	-	Posterior 1/3, Foramen Cecum	-
1983	Wasserstein, et al.	4, CR	50s, F	G/FB/Mass	-	-	Anterior 2/3	-
1975	McClendon	4, CS	Child, F	As	SSE, Oc, HC, MCB	Pedunculated	Posterior 1/3, Foramen Cecum	Excision
1975	McClendon	4, CS	20s, M	As	SSE, HC, MCB	-	Right Posterior 1/3	Excision
1971	Krolls, et al.	4, CS	20s, F	As	SSE, Oc, HC, Ob, MCB	-	Anterior 2/3	-
1971	Krolls, et al.	4, CS	20s, M	As	SSE, Oc, HC, Ob, MCB	Pedunculated	Posterior 1/3, Foramen Cecum	-
1971	Krolls, et al.	4, CS	70s, M	G/FB/Mass	SSE, Oc, HC, Ob, MCB	Pedunculated	Posterior 1/3	-
1971	Krolls, et al.	4, CS	Child, F	G/FB/Mass	SSE, Oc, HC, Ob, MCB	Pedunculated	Posterior 1/3, Foramen Cecum	-
1971	Krolls, et al.	4, CS	20s, F	As	SSE, Oc, HC, Ob, MCB	Sessile	Posterior 1/3	-
1971	Krolls, et al.	4, CS	Child, F	As	SSE, Oc, HC, Ob, MCB	Pedunculated	Right Posterior 1/3	-
1971	Krolls, et al.	4, CS	20s, M	As	SSE, Oc, HC, Ob, MCB	Sessile	Midline Anterior 2/3	-
1971	Krolls, et al.	4, CS	30s, M	As	SSE, Oc, HC, Ob, MCB	Pedunculated	Midline Anterior 2/3	-
1971	Goldberg, et al.	4, CS	60s, M	As	-	-	Anterior 2/3	-
1971	Begel, et al.	4, CS	20s, F	D	-	Sessile	Left Anterior 2/3	-
1968	Jahnke & Daly	4, CS	20s, F	G/FB/Mass	-	-	Midline Posterior 1/3	-
1968	Kaye	4, CS	20s, F	As	-	-	Posterior 1/3	-
1967	Cataldo, et al.	4, CS	30s, F	As	-	Pedunculated	Midline Posterior 1/3	-
1964	Church	4, CS	Child, F	G/FB/Mass	-	Pedunculated	Anterior 2/3, Foramen Cecum	-
1957	Klajman	4, CS	20s, F	As	-	-	Right Posterior 1/3	-
1956	Peimer, et al.	4, CS	20s, F	G/FB/Mass	-	-	Left Anterior 2/3	-
1950	Breckenridge & Lukens	4, CS	20s, F	As	-	Pedunculated	Right Anterior 2/3	-
1949	Witchell	4, CS	30s, F	G/FB/Mass	-	Pedunculated	Right Anterior 2/3	-
1938	Muta & Ogata	4, CS	20s, M	D	-	Pedunculated	Right Posterior 1/3, Foramen Cecum	-
1933	Magnien & Perrot	4, CS	30s, F	D	-	Sessile	Midline Posterior 1/3	-
1931	Jung	4, CS	20s, F	G/FB/Mass	-	Sessile	Anterior 2/3, Right and Left	-
1929	Zuckerman	4, CS	30s, F	G/FB/Mass	-	Pedunculated	Left Anterior 2/3	-
1925	Hirsch	4, CS	20s, F	D	-	-	Posterior 1/3	-
1913	Monserrat	4, CS	30s, F	As	-	Pedunculated	Posterior 1/3	-

**Abbreviations:** As, Asymptomatic; C, Cartilage; CR, Case Report; CS, Case Series; D, Dysphasia; G/FB/Mass, Gagging/Foreign Body Sensation/ Mass; H, Hoarseness; HC, Haversian Canals; LOE, Level of Evidence; MCB, Mature Compact Bone; Ob, osteoblasts; Oc, osteoclasts; Pain; S, Swelling; SSE, Stratified Squamous Epithelium;

## DISCUSSION

The purpose of this systematic literature review was to contribute an updated evaluation of published articles concerning osseous choristoma cases of the tongue. The authors hope that these results can help clinicians differentiate this type of lesion from other lingual masses and provide differential diagnosis guidance for when patients present with a lingual mass.

The 1971 systemic review that we cited earlier in this paper demonstrated a patient range from nine through 73 years of age.^5^ In subsequent case studies, patient ages have ranged from 5 to 73 years, showing how this pathology can affect patients of nearly any age.^13,14^ In our review, the age and gender distribution was skewed positively toward females in their 20s through later 30s. (Figure 2) When considering a differential diagnosis for more common lingual lesions (e.g., SCC), it may therefore be important to factor in both a patient’s age and gender when considering a possible osseous choristoma diagnosis.^5,12–14^

There remains limited information surrounding the etiopathology of osseous choristomas^5,15,16^ During the 1970s, there were two theories posed to explain the progression of this condition. One theory suggested that the development of osseous choristoma was often due to a congenital malformation.^17^ Another proposed theory was that lesions could arise from prior trauma or chronic irritation to the tongue.^17^ Currently, there remains little evidence supporting either theory.^18^

Previous reports have suggested that an osseous choristoma occurs more frequently in the dorsal portion of the tongue, often closely located along the circumvallate (vallate) papillae.^5^ Our findings were consistent with this earlier conclusion in that we found that 40 (58%) of our case lesions had occurred in the left, right, or midline of the posterior region of the tongue, with 15 (22%) of cases at the foramen cecum.

Our systematic review also revealed a wider size variation with masses as large as 2.5 cm. in diameter surpassing the 2.0 cm. in earlier reports.^5,12^ We also found masses being reported as small as 0.5 cm, consistent with the smallest 0.5 cm previously recorded.^5^ These lesions have historically demonstrated a hard mass with either pedunculated or sessile morphology.^5,18^ Notably, pedunculated masses with a narrower base is accommodating for routine surgical excision.

Of the cases we reviewed, 31 (44.9%) were asymptomatic, with the remaining 38 (55.1%) patients reported symptoms including dysphasia, gagging, foreign body sensation, mass effect, hoarseness, swelling, pain. (Figure 2) To understand the symptomatology of an osseous choristoma, it is necessary for the provider to consider the relevant clinical anatomy of the tongue to appreciate the impact that such a lesion can have on the patient’s oropharyngeal region.^5^

Diagnosis of an osseous choristoma is optimally confirmed following biopsy and histological testing. Once a diagnosis has been confirmed, surgical excision of an osseous choristoma under local anesthesia continues to be the most common treatment for the lesion.^16^ As noted during this review, some surgeons have used laser/CO_2_ as an alternative surgical technique.^13,14^

After surgical removal, none of the cases of lingual osseous choristoma in our systematic review exhibited recurrence. Previously documented cases of non-lingual osseous choristoma (e.g., gum or cheek locations), however, have exhibited recurrence up to 12 years after surgical removal.^19^ This suggests that although some forms of osseous choristomas may return, recurring oral masses may require a repeat biopsy is to clarify concurrent pathology.

## CONCLUSION

Osseous choristomas of the tongue are benign disease processes that are rarely diagnosed. Physicians should still consider this condition if patients present with a lingual mass and accompanying symptoms of a gagging/ globus sensation and pertinent lifestyles history, (e.g., chronic alcohol and tobacco use).^20,21^ Due to the complex anatomy of the tongue, it may be imperative to refer such patients to an otolaryngologist to remove such lesions.^21^ As medical researchers develop additional diagnostic and treatment modalities for osseous choriostomas, some form of surgical resection will continue to be the treatment mainstay to prevent worsening of symptoms and relapse.

### Conflict of interest

The authors declare no conflict of interest.

## References

[ref-35340] Neville Brad W., Damm Douglas D., Allen Carl M., Chi Angela C. (2019). Color Atlas of Oral and Maxillofacial Diseases.

[ref-35341] Gorini Edoardo, Mullace Mauro, Migliorini Luca, Mevio Emilio (2014). Osseous choristoma of the tongue: A review of etiopathogenesis. Case Reports in Otolaryngology.

[ref-35342] Macêdo Marina Barguil, Borges Saulo Evangelista Moura, Macêdo Plínio da Silva, Borges Paulo de Tarso Moura (2018). Lingual Osteoma as a fortuitous finding on a boy with post-adenoidectomy inflammatory pseudotumor. Oral and Maxillofacial Surgery Cases.

[ref-35343] Sapp J. PHILIP, Eversole LEWIS R., Wysocki GEORGE P. (2004). Contemp Oral Maxillofac Pathol.

[ref-35344] Krolls Sigurds O., Jacoway John R., Alexander William N. (1971). Osseous choristomas (osteomas) of intraoral soft tissues. Oral Surgery, Oral Medicine, Oral Pathology.

[ref-35345] Bouquot Jerry E., Muller Susan, Nikai Hiromasa (2009). Lesions of the Oral Cavity.

[ref-35346] Supiyaphun P., Sampatankul P., Kerekhanjanarong V., Chawakitchareon P., Sastarasadhit V. (1998). Lingual osseous choristoma: A study of eight cases and review of the literature. Ear Nose Throat.

[ref-35347] Rachappa M., Triveni M. (2010). Capillary hemangioma or pyogenic granuloma: A diagnostic dilemma. Contemp Clin Dent.

[ref-35349] Benamer M.H., Elmangoush A.M. (2007). Lingual osseous choristoma case report and review of literature. Libyan J Med.

[ref-35350] Liberati A., Altman D. G, Tetzlaff J., Mulrow C., Gotzsche P. C, Ioannidis J. P A, Clarke M., Devereaux P J, Kleijnen J., Moher D. (2009). The PRISMA statement for reporting systematic reviews and meta-analyses of studies that evaluate healthcare interventions: Explanation and elaboration. BMJ.

[ref-35351] (2020). OCEBM Levels of Evidence.

[ref-35352] Nash M., Harrison T., Lin P.T., Lucente F.E. (1989). Osteoma of the tongue. Ear Nose Throat J.

[ref-35353] West Charles B., Jr., Atkins James S., Jr. (1988). Choristomas of the intraoral soft tissues. Otolaryngology–Head and Neck Surgery.

[ref-35354] Cabbabe Edmond B., Sotelo-Avila Cirilo, Moloney Sean T., Makhlouf M. Vincent (1986). Osseous choristoma of the tongue. Annals of Plastic Surgery.

[ref-35355] Jeon Jae-Ho, Kim Min Gyun, Park Joo Yong, Lee Jong Ho, Kim Myung Jin, Myoung Hoon, Choi Sung Weon (2017). Analysis of the outcome of young age tongue squamous cell carcinoma. Maxillofacial Plastic and Reconstructive Surgery.

[ref-35356] Adhikari Bhoj Raj, Sato Jun, Morikawa Tetsuro, Obara-Itoh June, Utsunomiya Masafumi, Harada Fumiya, Chujo Takatoshi, Takai Rie, Yoshida Koki, Nishimura Michiko, Shakya Mamata, Nagayasu Hiroki, Abiko Yoshihiro (2016). Osseous choristoma of the tongue: Two case reports. Journal of Medical Case Reports.

[ref-35357] Engel P., Cherrick H.M. (1976). Extraosseous osteomas of the tongue. J Oral Med.

[ref-35358] Mortazavi Hamed, Safi Yaser, Baharvand Maryam, Rahmani Somayeh, Jafari Soudeh (2017). Peripheral Exophytic Oral Lesions: A Clinical Decision Tree. International Journal of Dentistry.

[ref-35359] Long Daniel E., Koutnik Alfred W. (1991). Recurrent intraoral osseous choristoma. Report of a case. Oral Surgery, Oral Medicine, Oral Pathology.

[ref-35360] Sunil A., Kurien J., Mukunda A., Bin Basheer A. (2013). Common superficial tongue lesions. Indian J Clin Pract.

[ref-35361] Reamy B.V., Derby R., Bunt C.W. (2010). Common tongue conditions in primary care. Am Fam Physician.

